# A network database for the human biobank

**DOI:** 10.1002/ggn2.10049

**Published:** 2021-05-27

**Authors:** Myles Axton

**Affiliations:** ^1^ Wiley Hoboken New Jersey USA

“Enduring trust is essential for lifelong collection of phenotypes and traits. Good metadata, local ownership and fair reuse of genomic and outcome data can be sustained in partnership among well‐resourced and well‐peopled regions of the world.“

A Perspective in this issue recounts the endeavors of the collaborative Virus Outbreak Data Access Network (VODAN)—Africa^2^ that collected SARS‐CoV‐2 outcomes using electronic case report forms (eCRFs) and other templates and organized the data with FAIR metadata models in linked data for clinical decision making and research queries. One use case employed temporal, numerical and geolocator metadata to connect de‐identified interviews with displaced people in Tunisia about their COVID‐19 infection outcomes to media reports that aggregate information on the same groups. The metadata model and the data it describes were stored as linked data that could be remotely queried across the network of nine African countries.

Genome data is cheap, plentiful, and concentrated in a few wealthy places, even relative to the information web, where less than 1% of the world's servers serve over 99% of the web content.^1^ This situation arises because it is difficult to move petabytes of data (since it is hard drives rather than bytes that travel). Second, the need for efficient search over similar formats of data usually leads to centralized accumulation of resources. Third, trusted and secure sharing of data resources among distributed sites requires metadata standards and linked data conventions that permit both computer operations without parsing or data transformation and queries from human users who range from clinicians to bioinformaticians to government agencies. Finally, application of any agreed metadata standards needs to be rapid and very low cost if it is to be more than a specialized research and training exercise.

In contrast to genome data, personal experiences including exposures and clinical records are distributed across institutions, homes, families, and individuals. Lifelong trust that sharing this information brings better outcomes for the donors is essential if we are to use this living biobank of diverse experience to make sense of variation in both viral and human genomes. Information from affected and unaffected individuals is needed to understand the importance of even point mutations in small viral genomes—such as the SARS‐CoV‐2 variants that continue to cause so much disruption and disease worldwide. Yet this data has not been gathered from places where the disruption is occurring, largely because we do not yet have collection networks with the trust and capacity to sustainably return results within the region of study.

There are now several related functional technologies for linked data to deliver the aspirational goals laid out in the principles of FAIR data and services. These working together would amount to a mercantile revolution in the global data trade rather than the gold rush metaphor of the Perspective.^2^ Shipping containers for ideas can be made from Research Object Crates^3^, ^4^ bearing just enough standard metadata for basic interoperability and relabeling. Unlike cargo, however, data will not move, but instead, the user's queries will travel systematically to data containers they identify by their appropriate licenses, permissions, provenance, and descriptions for use. Autonomously controlled pods of personal information can be licensed for social cooperation, research or profit as the owner sees fit.^5^, ^6^ This change of emphasis to good labeling for data visiting is the basis for developing products like a personal health train.^7^

The VODAN project has contributed to capacity building through its active interdisciplinary cooperation informatics training plan and has greatly promoted the cause of equitable autonomous and secure data ownership. However, it may be some time before the participating sites will be able both to innovate and interoperate fully on the same network in a distributed fashion as the project was originally conceived. Problems inherent in the stability of each local datastore's query protocol service and the potential for inadvertent divergence in implementation led to a tactical decision instead to use centrally provided metadata templates and mirrored storage at a secure central site (CEDAR). This redesign shows that central providers may gain sufficient trust, and partner through secure hosting and open commitment to the storage, organization and ownership of data by the participating local informatics communities across the network. The dream of building distributed capacity together across data rich and resource rich regions remains alive and compelling as ever.

## AUTHOR CONTRIBUTIONS

**Myles Axton:** Writing‐original draft; writing‐review & editing.

## Data Availability

No data availability statement in Editorial article format.
